# CRIPTO and miR-371a-3p Are Serum Biomarkers of Testicular Germ Cell Tumors and Are Detected in Seminal Plasma from Azoospermic Males

**DOI:** 10.3390/cancers12030760

**Published:** 2020-03-23

**Authors:** Cassy M. Spiller, João Lobo, Willem P. A. Boellaard, Ad J. M. Gillis, Josephine Bowles, Leendert H. J. Looijenga

**Affiliations:** 1 School of Biomedical Sciences, The University of Queensland, Brisbane, QLD 4072, Australia; c.spiller@uq.edu.au (C.M.S.); jo.bowles@uq.edu.au (J.B.); 2 Princess Máxima Center for Pediatric Oncology, Heidelberglaan 25, 3584 CS Utrecht, The Netherlands; jpedro.lobo@ipoporto.min-saude.pt (J.L.); A.J.M.Gillis@prinsesmaximacentrum.nl (A.J.M.G.); 3 Department of Pathology, Portuguese Oncology Institute of Porto (IPOP), R. Dr. António Bernardino de Almeida, 4200-072 Porto, Portugal; 4 Cancer Biology and Epigenetics Group, IPO Porto Research Center (GEBC CI-IPOP), Portuguese Oncology Institute of Porto (IPO Porto) & Porto Comprehensive Cancer Center (P.CCC), R. Dr. António Bernardino de Almeida, 4200-072 Porto, Portugal; 5 Department of Pathology and Molecular Immunology, Institute of Biomedical Sciences Abel Salazar, University of Porto (ICBAS-UP), Rua de Jorge Viterbo Ferreira no. 228, 4050-313 Porto, Portugal; 6 Department of Urology, Erasmus MC Cancer Institute, University Medical Center, 3015 GD Rotterdam, The Netherlands; w.boellaard@erasmusmc.nl; 7 Department of Pathology, Lab. for Exp. Patho-Oncology (LEPO), Erasmus MC-University Medical Center Rotterdam, Cancer Institute, Be-432A, PO Box 2040, 3000 CA Rotterdam, The Netherlands

**Keywords:** CRIPTO, fertility, germ cells, germ cell tumors, miR-371a-3p, semen, serum, testicular cancer

## Abstract

miR-371a-3p is currently the most informative reported biomarker for germ cell tumors (GCTs). Another developmental-related biomarker, CRIPTO, is involved in the regulation of pluripotency and germ cell fate commitment. We aimed to assess the value of CRIPTO as a diagnostic and prognostic biomarker of testicular GCTs (TGCTs) and also to assess its presence in seminal plasma samples, compared with miR-371a-3p. In total, 217 and 94 serum/seminal plasma samples were analyzed. CRIPTO was quantified using ELISA and miR-371a-3p using bead-based isolation followed by RT-qPCR. Methylation profiling (EPIC array) for the CRIPTO promoter region was undertaken in 35 TGCT tissues plus four (T)GCT cell lines. Significantly higher CRIPTO concentration was found in sera of non-seminomas compared to controls (*p* = 0.0297), and in stage II/III disease compared to stage I (*p* = 0.0052, *p* = 0.0097). CRIPTO concentration was significantly positively correlated with miR-371a-3p levels in serum (*r* = 0.16) and seminal plasma (*r* = 0.40). CRIPTO/miR-371a-3p levels were significantly higher in seminal plasma controls when compared to serum controls (*p* = 0.0001, *p* < 0.0001). CRIPTO/miR-371a-3p were detected both in normospermic and azoospermic males, and levels were higher in TGCTs compared to GCNIS-only. We have provided the largest dataset of evaluation of CRIPTO in serum and seminal plasma of GCTs, showing its potential value as a biomarker of the disease.

## 1. Introduction

Germ cell tumors (GCTs) are developmental cancers since they recapitulate the various steps of embryonic and germ cell development [1,2]. They can arise in both the testes and ovaries and also in extragonadal topographies, reflecting the migration of nascent germ cells along the midline of the body towards the gonads [1]. Among GCTs, the type II GCTs of the testis (TGCTs) are by far the most numerous and clinically challenging. These postpubertal-type tumors are among the most common solid neoplasms in adolescent and young-adult Caucasian men [3], and derive from primordial germ cells/gonocytes that are arrested in their maturation, giving rise to a precursor lesion called germ cell neoplasia in situ (GCNIS) [4], a central concept of the most recent World Health Organization Classification for these tumors [5]. From GCNIS emerges the most common subtype―the seminoma (SE)―and, after a reprogramming process takes place, the various non-seminoma (NS) histologies―including embryonal carcinoma (EC), yolk sac tumor (YST), choriocarcinoma (CH), and teratoma (TE) [5,6].

This developmental perspective for TGCT origin and progression has led to the discovery of several biomarkers of the disease, both tissue-based (pluripotency factors, such as OCT3/4, SOX2, and SOX17) and liquid biopsy-based (including the ‘classical’ serum markers alpha fetoprotein (AFP) and human chorionic gonadotropin subunit beta (β-HCG)). Better non-invasive liquid-biopsy biomarkers are needed to overcome the limitations of the classical markers, which are overall elevated in only about 60% of patients at the time of diagnosis [7,8]. In the last decade, embryonic microRNAs of the 371~373 cluster (especially miR-371a-3p) have proved their value, outperforming the classical markers in various clinical settings [9].

Another biomarker related to germ cell development is CRIPTO, also known as teratocarcinoma derived growth factor 1, TDGF1. It is an obligate co-receptor of NODAL, a member of the TGF-β signaling family, which has determinant roles in the process of commitment to the male germ cell fate, in embryogenesis, and in stem cell pluripotency more broadly [10,11,12,13]. The NODAL/CRIPTO signaling pathway is believed to regulate the delicate balance between the proliferation of germ cells and fate commitment, meaning that disturbance of this signaling cascade may lead both to infertility (due to insufficient spermatogonial stem cells) or to prolonged pluripotency, resulting in the development of TGCTs [14]. This is in line with the finding of ectopic NODAL/CRIPTO signaling activation in NS [15]. CRIPTO was also found to be at least partially regulated by promoter methylation [16,17]. As has been found for other neoplasms [18], we previously showed, in a small set of samples, that CRIPTO is detectable in serum of TGCT patients [16]. This result suggested that CRIPTO could constitute a non-invasive diagnostic marker of the disease, particularly if a sensitive assay can be developed. Moreover, and given the proximity of this bodily fluid to the testis, we hypothesized that CRIPTO detection in semen could prove a sensitive biomarker of TGCTs and a good surrogate to indicate the germ cell status of male patients, as was recently suggested for miR-371a-3p [19,20].

In this work, we assessed the value of CRIPTO as a serum diagnostic and prognostic biomarker of TGCTs, expanding our previous series, and assessed the sensitivity of detection in seminal plasma samples of patients with both TGCTs and infertility. Finally, we correlated our findings with the levels of miR-371a-3p detected in these bodily fluids.

## 2. Results

### 2.1. Blood Serum Samples

#### 2.1.1. CRIPTO and miR-371a-3p Detection of TGCT Subtypes

**Figure 1:**
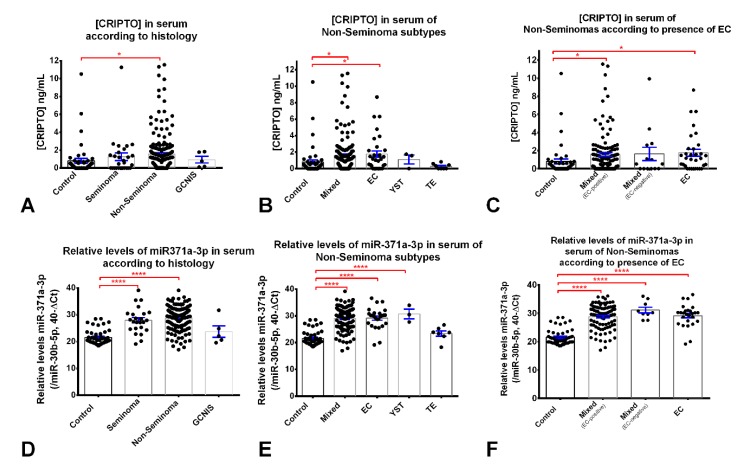
CRIPTO concentration and miR-371a-3p relative levels in the serum cohort, according to histological group. CRIPTO concentration (ng/mL) assessed by ELISA ((**A**) Kruskal–Wallis test *p*-value = 0.0232) and miR-371a-3p relative levels assessed by RT-qPCR ((**D**), Kruskal–Wallis test *p*-value < 0.0001) in serum of controls (healthy blood donors, *n* = 48), seminoma (*n* = 27), non-seminoma (*n* = 190) and GCNIS-only patients (*n* = 5); in serum of controls and the various non-seminoma subtypes ((**B**,**E**) Kruskal–Wallis test *p*-values of 0.0055 and <0.0001); and in serum of controls and non-seminoma patients with and without EC ((**C**,**F**) Kruskal–Wallis test *p*-values of 0.0111 and <0.0001). Error bars represent mean ± S.E.M. Relative levels of miR-371a-3p were normalized with miR-30b-5p and plotted in 40-ΔCt format. Abbreviations: EC—embryonal carcinoma; GCNIS—germ cell neoplasia in situ; YST—yolk sac tumor; TE—teratoma; S.E.M.—standard error of mean. * *p* < 0.05; **** *p* < 0.0001.

CRIPTO concentration in the serum sample cohort is depicted in Figure 1A. There were 184 positive samples: 30 controls, 15 SE, 134 NS, and 5 GCNIS-only. CRIPTO concentration was significantly higher in NS when compared to controls (*p* = 0.0297, Table 1, Figure 1A). No significant differences were observed between SE and NS, nor between controls and the five GCNIS-only patients. After separating the NS samples into the various histological subtypes, pure EC showed the highest CRIPTO concentration, followed by mixed tumors, both significantly higher than in controls (*p* = 0.0331 and *p* = 0.0309, Figure 1B, Table 1). After further separating the mixed tumors into EC-presence/absence, we found that those containing EC components had significantly higher CRIPTO concentration than controls (*p* = 0.0315, Figure 1C, Table 1).

Regarding promoter methylation analyses, all (T)GCT cell lines displayed similar promoter methylation beta values (between 0.75 and 0.86, Supplementary Figure S1A); regarding tumor samples, the highest methylation levels (mean beta values) were observed for EC (both in primary and metastatic tumors, 0.64 and 0.67), and the lowest for YST (0.49) and TE (0.26) (Supplementary Figure S1B,C).

Relative levels of miR-371a-3p are plotted in Figure 1D. Significantly higher relative levels of miR-371a-3p were seen in NS and in SE when compared to controls (*p* < 0.0001 for both). No significant differences were observed between SE and NS. miR-371a-3p relative levels did not differ significantly between controls and the five GCNIS-only patients (Figure 1D). Again, after separating these data into the various NS histologies, pure EC, mixed tumors and YST showed significantly higher relative levels of miR-371a-3p compared to controls (*p* < 0.0001 for all comparisons, Figure 1E); there was no significant difference between TE and controls (Figure 1E). Both EC-positive and EC-negative mixed tumors showed significantly higher miR-371a-3p relative levels when compared to controls (Figure 1F).

**Figure 2:**
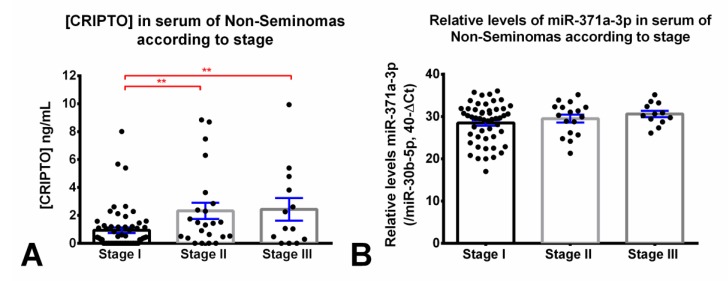
CRIPTO concentration and miR-371a-3p relative levels in the non-seminoma serum cohort, according to disease stage. CRIPTO concentration (ng/mL) assessed by ELISA ((**A**) Kruskal–Wallis test *p*-value = 0.0121 and 0.4250) and relative levels of miR-371a-3p assessed by RT-qPCR (**B**) in non-seminoma patients with stage I, II, and III disease. Error bars represent mean ± S.E.M. Relative levels of miR-371a-3p were normalized with miR-30b-5p and plotted in the 40-ΔCt format. ** *p* < 0.01.

Among NS patients with available staging (*n* = 94), CRIPTO concentration was significantly higher in samples from patients with stage II and stage III disease when compared to stage I (*p* = 0.0052 and *p* = 0.0097, respectively, Figure 2A, Table 3). CRIPTO was detected in 100% of stage III patients (13 total) and in 23 (39.7%) and 9 (39.1%) of stage I and II, respectively (Table 2).

Relative levels of miR-371a-3p increased with disease stage in the NS group (stage I: mean 28.5; stage II: mean 29.5; stage III: mean 30.6), although this trend did not prove to be statistically significant (Figure 2B).

AFP levels were not significantly higher in stage II or III disease compared to stage I (Supplementary Figure S2A), but β-HCG levels in stage III disease were significantly higher than in stage I patients (Supplementary Figure S2B). The proportion of AFP-positive and β-HCG-positive cases was higher in stage III disease, but the difference did not achieve statistical significance (Supplementary Figure S2C,D).

#### 2.1.2. Correlation between CRIPTO Concentration and miR-371a-3p Levels in Serum

**Figure 3:**
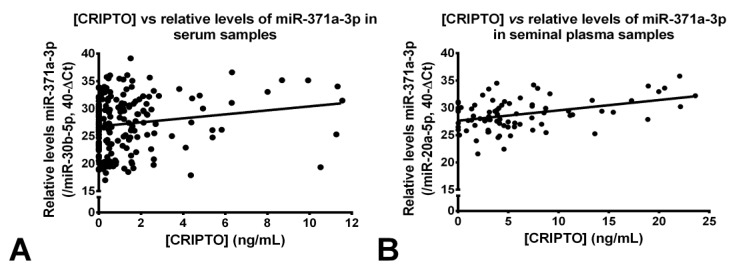
Correlation between CRIPTO concentration and miR-371a-3p relative levels in the serum samples (**A**) and in the seminal plasma samples (**B**). Relative levels of miR-371a-3p were normalized with miR-30b-5p (serum) and miR-20a-5p (seminal plasma) and plotted in the 40-ΔCt format.

We next assessed whether there was a correlation between CRIPTO concentration and relative levels of miR-371a-3p in matched sera samples representing all subgroups (controls, NS, SE, GCNIS-only). There was a significant, although weak, positive correlation between CRIPTO concentration and the relative levels of miR-371a-3p in matched serum samples (*r* = 0.16, *p* = 0.025, Figure 3A).

#### 2.1.3. Correlation between CRIPTO Concentration/miR-371a-3p Levels and Duration of Sample Storage

Because our sera sample cohort spanned 18 years of collections, we investigated whether time in storage bore any correlation with CRIPTO concentration and miR-371a-3p levels. There was a significant, although weak, positive correlation between CRIPTO concentration and the time of storage of the NS sera samples (*r* = 0.18, *p* = 0.0150, Supplementary Figure S3A). This correlation became non-significant (*r* = 0.14, *p* = 0.0797) when we restricted the sample set to 14 years post-collection. For the miR-371a-3p, however, no such correlation was found for samples collected over the total 16 years assessed (*r* = 0.06, *p* = 0.4509, Supplementary Figure S3B).

### 2.2. Seminal Plasma Samples

#### 2.2.1. CRIPTO and miR-371a-3p Detection in Serum vs. Seminal Plasma of Male Controls

**Figure 4:**
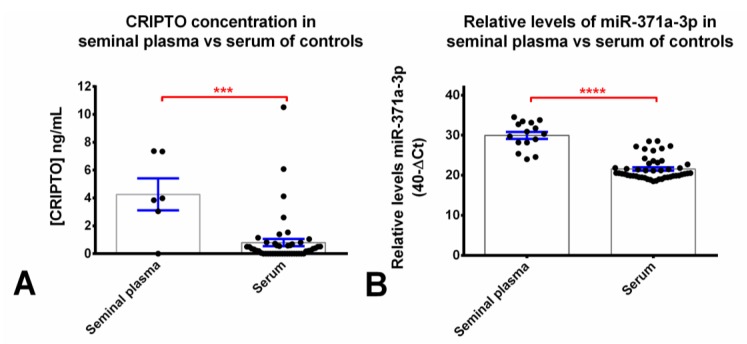
Comparison of CRIPTO concentration (**A**) and miR-371a-3p relative levels (**B**) between serum and seminal plasma male controls. Relative levels of miR-371a-3p were normalized with miR-30b-5p (serum) and miR-20a-5p (seminal plasma) and plotted in the 40-ΔCt format. Error bars represent mean ± S.E.M. *** *p* < 0.001, **** *p* < 0.0001.

We hypothesized that seminal plasma would yield higher sensitivity and specificity of detection of TGCT-relevant biomarkers due to proximity to the tumor cells of origin. Seminal plasma samples of male controls exhibited significantly higher CRIPTO concentration when compared to the pool of sera samples from male controls (5.4 times higher, *p* = 0.0001, Figure 4A, Table 3). The same was observed for relative levels of miR-371a-3p (*p* < 0.0001, Figure 4B).

#### 2.2.2. CRIPTO and miR-371a-3p Detection in Azoospermic Patients

**Figure 5:**
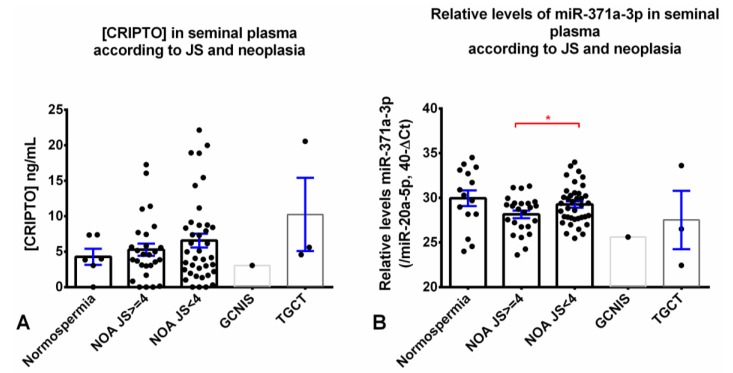
CRIPTO concentration ((**A**) Kruskal–Wallis test *p*-value = 0.5632) and miR-371a-3p relative levels ((**B**) Kruskal–Wallis test *p*-value = 0.1495) in various seminal plasma samples. Comparison of different samples, including those from men with normospermia, non-obstructive azoospermia with different Johnsen’s scores, and with GCNIS/TGCT. Relative levels of miR-371a-3p were normalized with miR-20a-5p and plotted in the 40-ΔCt format. Error bars represent mean ± S.E.M. Abbreviations: JS—Johnsen’s score; NOA: non-obstructive azoospermia; GCNIS - germ cell neoplasia in situ; TGCT—testicular germ cell tumor. * *p* < 0.05. For CRIPTO testing, only six seminal plasma samples were available, while 15 were available for miR-371a-3p testing.

We detected CRIPTO in all seminal plasma sample groups, including normospermic controls (5/6, 83.3%) and azoospermic males (72/78, 92.3%). Our assay detected CRIPTO both in non-obstructive azoospermia (62/67, 92.5%) and in epididymal obstructive azoospermia (10/10, 100%), but was negative in the single structural obstruction (CBAVD) sample with enough material for CRIPTO assessment (Table 4). There were no significant differences in CRIPTO concentration among non-obstructive azoospermic samples from patients with Johnsen’s score lower (only pre-meiotic germ cells) or higher than four (presence of post-meiotic germ cells, Figure 5A), although samples with lower Johnsen’s score displayed higher readings (mean 6.6 ng/mL vs. 5.3 ng/mL, Table 4).

Relative levels of miR-371a-1p were high in cases of normospermia (mean 30.0) and progressively lower in non-obstructive azoospermia, epididymal obstructive azoospermia, and the two CBAVD cases (mean 28.8, 28.3, and 25.7, respectively), although differences did not reach significance. Non-obstructive azoospermic males with lower Johnsen’s score showed significantly higher relative levels of miR-371a-3p when compared to those with high Johnsen’s score (*p* = 0.0497, Figure 5B).

In the four azoospermic males that had simultaneous evidence of TGCT/GCNIS, levels of CRIPTO and miR-371a-3p did not differ overall from the distribution in the remaining azoospermic males, nor the normospermia controls; these sample numbers are too low for achieving any definitive conclusion, but show that both biomarkers can be detected in these patients as well.

**Figure 6:**
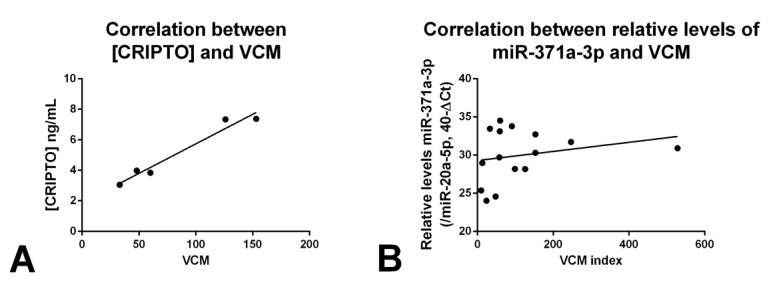
Correlation between volume × concentration × % motility (VCM) index and CRIPTO concentration (**A**) and miR-371a-3p relative levels (**B**) in seminal plasma samples of normospermic males. For CRIPTO, the five CRIPTO-positive samples are plotted (the remaining one was CRIPTO-negative). Relative levels of miR-371a-3p were normalized with miR-20a-5p and plotted in the 40-ΔCt format. Abbreviations: VCM—volume × concentration × % motility.

Several parameters are used to assess semen quality, including volume, concentration, and motility, and these traditional variables allow for an accurate and informative evaluation [21]. The total motile sperm count (volume × concentration × % motility; VCM) is frequently used for comparing data among studies [22]. Considering the control seminal plasma samples where CRIPTO was detected, we found a significant and strong positive correlation between CRIPTO concentration and total motile sperm count (*r* = 0.98, *p* = 0.004, Figure 6A). Although a weak positive correlation was also found for miR-371a-3p, it did not reach statistical significance (*r* = 0.23, *p* = 0.409, Figure 6B).

#### 2.2.3. Correlation between CRIPTO Concentration and miR-371a-3p Levels in Seminal Plasma

There was a significant positive correlation between CRIPTO concentration and the relative levels of miR-371a-3p in matched seminal plasma samples (*r* = 0.4, *p* = 0.0002, Figure 3B). This correlation in seminal plasma was greater than that observed in blood serum (*r* = 0.16, Figure 3A).

## 3. Discussion

The biology of GCTs closely relates to developmental germline events; their emergence results from defective programming in their cells of origin―the primordial germ cells (PGCs) [1]. During this period of vulnerability during fetal life, disturbances in the germ cell niche that alter PGC differentiation can lead to arrest in maturation and development of the precursor lesion GCNIS. GCNIS cells are the ‘cancer stem cells’ of the testis and give rise to the various TGCT subtypes after puberty [23]. Despite a report of infrequent CRIPTO expression in urological malignancies (including only four testicular tumors) [24], more recently we and others have described the high expression of CRIPTO in TGCTs and related cell lines (with a decreased expression upon exposure to differentiation-inducing agents, such as retinoic acid), and hypothesized that aberrant maintenance or re-activation of NODAL/CRIPTO signaling during fetal life may be a possible mechanism for the genesis of these tumors [14,16,25,26]. The role of CRIPTO during fetal germline development is linked strongly to pluripotency of these cells in mice [14], and we have also shown it to be expressed in the spermatogonial stem cells of the adult human testis [16]. NODAL signaling, which is facilitated by CRIPTO, has also been implicated in the maintenance of normal somatic testis development in humans [11]. In this work, we aimed to explore the expression of CRIPTO in liquid biopsy samples of patients with TGCTs and of infertile males, extending our previous work [16] and providing novel insight on the role of this pathway in regulating this balance between tumor development and infertility. We additionally aimed at correlating our findings with the relative levels of miR-371a-3p, the most promising TGCT liquid biopsy biomarker identified to date [27].

In this study, significantly higher CRIPTO concentrations were detected in serum samples from NS patients when compared to controls, and readings were higher than for the SE patient cohort, although this was not statistically significant (Figure 1A). The highest mean concentration of CRIPTO was found in patients harboring pure EC (1.76 ng/mL). These results are in line with our previous analyses where, using RT-qPCR and immunohistochemistry, we found the highest CRIPTO expression in EC (and YST) compared to other subtypes and controls, which was the reason for enriching our cohort on NS samples, together with the need to represent the various subtypes within this heterogeneous group of tumors [16]. In our previous ELISA assessment of a small set of TGCT patient sera, we found the highest mean CRIPTO concentration in SE, and a reading for only 1/15 controls (6% positivity) [16]. This discrepancy is likely accounted for by the small cohort of 44 tumors and 15 controls assessed initially compared to this larger study, comprising 270 tumors and 48 controls. Further, our current assay had greater sensitivity as we achieved 63% positive CRIPTO readings (background levels) in our control serum cohort (compared to 6% in the small study).

We note in our current analyses at least one obvious outlier in the control blood sera group with a very high CRIPTO concentration (10.52 ng/mL). If this one sample is removed, the overall mean CRIPTO control concentration is decreased, and statistical significance is increased for many of our subgroup analyses (as presented in Supplementary Table S1). Although ‘control’ individuals were blood donors presumed to be healthy at the time of sample collection, we hypothesize that this outlier could be due to the presence of an undiagnosed tumor (somatic or TGCT) or other condition; unfortunately, this possibility cannot be excluded nor verified due to the anonymized dataset. Further enlargement of the control group to replicate natural interindividual variation is desirable. Our control group CRIPTO concentration mean of 0.79 ± 0.29 ng/mL (or 0.59 ± 0.16 ng/mL with the highest outlier removed) is similar to the control mean of Pilgaard et al. [28] of 0.60 ng/mL, using the same assay.

For a diagnostic assay to be useful in the clinical setting, it must be specific and sensitive. Our assay detected 56% of NS and 48% of SE patients and had a false positive rate (in controls, outlier included) of 21% using a control CRIPTO concentration cutoff set to 0.79 ng/mL. This sensitivity was further increased to 57% NS and 52% of SE and with a false positive rate (in controls, outlier included) of 29%, using the lower cutoff of 0.59 ng/mL. These results are approaching those of Pilgaard and colleagues who detected 70% of new glioblastoma multiforme cases using this assay [28] and are an improvement on our previous smaller analysis where we detected 36% of GCTs [16]. Within the NS tumor cohort, CRIPTO concentration out-performed classical serum markers AFP and β-HCG for the indication of disease stage (stage I vs. II and III; Figure 2A, Table 2), in line with the findings that the highest CRIPTO expression in glioblastoma patients (ELISA) [28] and esophageal squamous cell carcinoma (immunohistochemistry) [29] correlates strongly with poorer survival prognosis. 

Detection of GCNIS in individuals prior to tumor development is the ultimate prognostic/diagnostic goal to reduce the need for chemotherapy and radiation treatments and improve overall GCT patient survival. In our small GCNIS-only patient group, we detected 40% (2/5) of patients harboring GCNIS using the higher CRIPTO cutoff of 0.79 ng/mL, and this was further increased to 60% (3/5) of patients using the lower cutoff of 0.59 ng/mL. The assessment of larger studies that include validated tumor-free controls would help in setting the optimal CRIPTO cutoff value to more accurately determine tumor presence and possibly stage of severity. A larger sample cohort for GCNIS-only patients is also required to determine whether CRIPTO is a sufficiently specific and sensitive prognostic marker for this pre-cancerous condition. Further assay optimization, using alternate CRIPTO antibodies and concentrations, as well as possibly other assay platforms (such as ELISA-qPCR or multiplexing with other targets), may aid in increasing specificity and sensitivity to clinically useful levels. Relevant to this purpose, our analyses indicated a need for reasonably prompt determination of CRIPTO concentration in serum samples since slightly higher readings were found in serum samples stored for long periods (Supplementary Figure S3A).

It is logical to assume that seminal plasma would be of higher relevance than blood sera for detecting specific testicular malignancies, including the presence of pre-cancerous GCNIS alone, given the closer proximity to the tumor/GCNIS cells of origin. Although we assessed CRIPTO concentration in a small cohort (*n* = 6) of control (normospermic) seminal plasma samples, we found the mean concentration to be five-times greater than that in the blood sera control group (Figure 4, Table 3). This finding of higher basal levels of CRIPTO in semen is likely due to (1) endogenous CRIPTO expression in the spermatogonial stem cells (SSCs) of the human testis [16], and (2) the closer proximity to the cells of origin (SSCs), without the need for the biomarker to cross the blood–testis barrier. Such higher basal readings may be more clinically useful, due to higher sensitivity (lower false-negative rate; we detected CRIPTO in 92% of the total cohort of seminal plasma samples, compared to 68% of the sera total cohort). This discovery also suggested that CRIPTO should be investigated as a fertility biomarker (independent of malignancies, which was the primary aim of our study).

Within our seminal plasma dataset of azoospermic men (which was our experimental setting for studies in this biofluid), we came across four patients with TGCT/GCNIS and tested them for these markers as well. Although the low number of samples does not allow us to establish any conclusion, we describe that CRIPTO can be detected in the seminal plasma of these patients. In this preliminary impression, the three tested TGCT cases showed higher baseline CRIPTO concentrations (10.24 ng/mL) than controls (4.26 ng/mL), while the GCNIS-only male showed levels comparable to controls. It is clear that a much larger dataset of controls, TGCT, and GCNIS-only samples is needed in order to confirm whether or not CRIPTO would be a useful cancer biomarker in this fluid, as we have suggested for blood sera.

To investigate the relevance of CRIPTO concentration to fertility, we assessed this in a relatively large and well-defined seminal plasma cohort (*n* = 79) of azoospermic males. Despite the absence of spermatozoa, CRIPTO was detected at levels similar to controls for non-obstructive azoospermia with JS ≥ 4, and at higher average levels for non-obstructive azoospermia with JS < 4 (not statically significant; Figure 5A). Further, CRIPTO was detected in non-obstructive and also in clinically assumed obstructive (epidydimal) azoospermia, but not in the single structural obstruction (CBAVD) sample. This finding is likely due to the fact that our ELISA detects the smaller cleaved portion of the CRIPTO protein that is shed from the cell surface (presumably the SSCs), rather than relying on the local presence of the cells of origin. The trend for higher CRIPTO with the lower JS may reflect the skewed abundances of cell populations in the testis: reduction/loss of differentiated spermatozoa (low JS; negative for CRIPTO) biases the total population towards undifferentiated (CRIPTO positive; SSCs) cells. Seemingly counterintuitive to this argument, however, we found that CRIPTO was strongly and positively correlated with the VCM index (Figure 6). Yet, in this instance, it is important to note that this correlation existed within the control group, which had an overall lower average CRIPTO concentrations than the non-obstructive azoospermia and TGCT sample groups. The reason for detection of CRIPTO in the epididymal obstructive azoospermia cases is still elusive, but we hypothesize it might be due to incomplete obstruction allowing passage of the cleaved CRIPTO protein despite no passage of sperm cells. Alternatively, another origin, like the seminal vesicles, must be kept in mind.

Finally, in this study, we also re-visited previous CRIPTO methylation analyses using a new technology platform. We have previously shown by direct sequencing that the methylation levels of two CpG regions of the CRIPTO promoter were low in human (T)GCT cell lines and did not correlate with CRIPTO gene expression [16]. In this study, using an EPIC methylation array, we found similar methylation readouts among the same four (T)GCT cell lines (representing SE and NS; Supplementary Figure S1A), although overall levels were higher than previously determined (mean beta values between 0.75–0.86). In a previous analysis of TGCT tissue biopsy samples, we found a correlation between promoter methylation and gene expression for certain subtypes (EC, TE, and CH), although we failed to do so for others (YST and SE) [16]. In this current study, we confirmed differential methylation levels among histological subtypes (Supplementary Figure S1B,C), as described by Costa et al. [17] (which showed higher CRIPTO methylation in NS). However, there was no clear correlation with CRIPTO concentration in blood serum (for example, methylation levels are relatively high in EC (Supplementary Figure S1B), the subtype that also shows the highest average CRIPTO concentration, Figure 1B). Differences in the specific promoter regions investigated between the two studies likely account for some of the discrepancies we are reporting here, although we can conclude that methylation of the CRIPTO promoter may be partially, but not completely, regulating CRIPTO gene expression in GCTs.

Because the microRNA miR-371a-3p is the most informative TGCT serum marker known to date, we were very interested to compare relative miR-371a-3p expression with our CRIPTO concentration dataset in both sera and semen. Assessment of over 80% of the same sera samples for miR-371a-3p revealed a weak, although positive, and significant correlation between the two biomarkers (Figure 3A), and this correlation was stronger in the total 84 seminal plasma samples assessed for both (Figure 3B), perhaps due to closer proximity of this bodily fluid to the testis’ source and, therefore, higher (and more sensitive) overall readings (as discussed above).

As has been reported previously, we confirmed robust and reliable detection of the NS and SE malignancies using miR-371a-3p expression relative to controls [30]. Similarly to CRIPTO, both TE and GCNIS-only samples were not significantly different from controls (as reported previously [31]), however, using the baseline cutoff of 21.55, it detected 92.2% of NS, 91.7% of SE, 60.0% of GCNIS-only, and 85.7% of TE (Figure 1D–F). In our particular sample cohort, and unexpectedly, we were unable to detect a significant difference between NS tumor stages I, II, and III, as had been reported previously [32]. We hypothesize this can be due to the limited number of samples, specific composition of the cohort, and possibly due to some cases of the overstaging of stage II TGCTs based on imaging criteria. However, our dataset did reveal that long periods in storage (up to 16 years) did not significantly influence readings, highlighting another versatility of miR371a-3p as a serum biomarker (Supplementary Figure S3B).

Assessment of miR-371a-3p in the same azoospermic seminal plasma cohort in which we assessed CRIPTO concentration similarly revealed higher basal levels than in blood sera (as had been reported previously [20,33]) and levels not significantly differing from normospermic males (Table 4, Figure 5B). We did detect miR-371a-3p in the obstructive azoospermia and non-obstructive azoospermia cohorts, as well as the TGCT cohort and single GCNIS-only sample, which is similar to a previous report [20]. In the non-obstructive azoospermia samples, we also found the highest average miR-371a-3p reading in the group with the lower Johnsen’s score; we hypothesize this may be due to a cell-type dilution effect as we described above for CRIPTO. While we expected to detect CRIPTO in non-obstructive azoospermic seminal plasma due to its ability to be cleaved and be expelled from the testis independent of any spermatozoa, it is still unclear how miR-371a-3p might also be detectable in this fluid. Since it was detected in non-obstructive azoospermic men, we hypothesize that SSCs may also be the source of miR-371a-3p; again, its detection in obstructive azoospermia samples (although in lower levels) deserves further investigation in future studies, and may indicate some contribution from the seminal vesicles.

Relative levels of miR-371a-3p have been shown to be positively correlated with sperm concentration [19,20], so it was suggested that this microRNA could function as a surrogate of germ cell composition/niche in males. In this work, as for CRIPTO, we found a positive correlation between miR-371a-3p and the VCM index (although not statically significant; Figure 6). Such a correlation may be due to spermatozoa harboring miR-371a-3p as has been suggested [20] as opposed or in addition to, immature germ cells/SSCs also expressing the microRNA [4]. Our previous work has suggested that the source of miR-371a-3p in semen samples is the germ cell compartment [19], with detection in testicular parenchyma and semen, but not in other urogenital tract locations.

Limitations of our work include its retrospective nature, and especially the limited number of samples in certain categories. Our preliminary data describing levels of CRIPTO and miR-371a-3p in seminal plasma samples of TGCT/GCNIS patients indicate that these biomarkers can be detected in these patients, but much larger studies are needed to conclude on the usefulness of these biomarkers in this context. This includes a large sample set of adequate normospermic controls and patients with TGCT particularly. For sera samples, more age-matched controls and GCNIS-only patient samples should be assayed, to conclude definitely on the usefulness of these markers for its detection, together with optimal storage and processing of samples. These future studies, ongoing in our Institute, will allow us to better set the relevant cutoffs according to the tested body fluid, and also to refine the ELISA assay for CRIPTO.

## 4. Materials and Methods

### 4.1. Patients and Samples

A total of 217 serum samples from TGCT patients (190 NS and 27 SE) and five from GCNIS-only patients were available for inclusion in the study. Samples were collected from patients in several centers across The Netherlands between 2001–2018 immediately before an orchiectomy procedure, and processed and stored at −80 °C. Additionally, a set of serum samples from male blood donors was collected (*n* = 48, provided by Sanquin, Rotterdam), achieving a total of 270 serum samples overall suitable for testing. A detailed clinical description of this serum cohort is presented in Table 5. Median age at diagnosis of the TGCT patients was 29 years (interquartile range 25–39); although significantly lower than in the control group, there was no significant correlation between age of controls/TGCT patients and CRIPTO levels (*p* = 0.750, *r*_s_ = −0.47; *p* = 0.112, *r*_s_ = −1.44) or miR-371a-3p levels (*p* = 0.754, *r*_s_ = −0.46; *p* = 0.528, *r*_s_ = 0.62). Most tumors were NS (*n* = 190, 87.6%), and among these the majority were mixed tumors (*n* = 147, 77.4%). There were 27 SE; however, one patient displayed remarkably high serum AFP levels at diagnosis and was clinically treated as an NS. The majority (*n* = 58, 61.7%) of cases were stage I.

Importantly, all the 270 samples in the study were tested for CRIPTO (as indicated in Table 1), while only 217 of these had enough sample left and could be additionally tested for miR-371a-3p. Information about tumor staging could be retrieved in total for 94/190 NS patients; hence, analyses on tumor stage for CRIPTO refer only to this pool of 94 samples (Table 2). Within the set of samples tested for miR-371a-3p, 80 samples had staging available (Figure 2B).

A total of 94 semen samples donated by males attending the Erasmus MC for andrological work-up were available for inclusion in the study. Semen samples were collected by masturbation after three to five days of abstinence. All samples were allowed to liquefy at 37 °C for 60 minutes before analysis, as per the World Health Organization 2012 criteria. Volume × concentration × % motility was recorded (“VCM index” or total motile sperm count) for normospermic individuals. Semen was then processed (to achieve the seminal plasma fraction) and stored at −80 °C. After thawing, these samples were used for ELISA and microRNA isolation (described below). Clinical details of the seminal plasma sample cohort are depicted in Table 6. Samples corresponded to normospermic males (*n* = 15) and azoospermic males (*n* = 79). The latter included proven non-obstructive azoospermia (*n* = 67), obstructive azoospermia (proven epididymal obstruction, *n* = 10), and those with obstructive azoospermia due to congenital bilateral absence of vas deferens (i.e., CBAVD) (*n* = 2). Included in the cohort were three samples from patients with a TGCT (two NS and one SE) and one sample from a patient with GCNIS-only, as detailed in Table 6 (footnotes). In addition, three of the non-obstructive azoospermic males had an *AZF* deletion (Table 6, footnotes). The median age at the time of collection of the sample was 38 years for the normospermia group and 35 years for the azoospermia group. Testicular sperm extraction (TESE) was performed in all 79 azoospermic males, finding sperm in 46 patients (58.2%). Most azoospermic patients had a Johnsen’s score of 2—Sertoli cell-only (41/79, 51.9%).

Due to constraints in sample availability, of the whole cohort described, 92/94 samples were tested for the miR-371a-3p, and only 84/92 were tested for CRIPTO (as depicted in Table 4 and plotted in Figure 5, which present the specific numbers included in each subgroup analyses). Specifically, the main difference is due to the normospermia samples, as only 6/15 of these could be assayed for CRIPTO, while all 15 available normospermia samples in the study were tested for miR-371a-3p (as plotted in Figure 4). Moreover, of the two CABVD samples, only one could be tested for CRIPTO, while both had material enough for miR-371a-3p detection.

Thirty-five TGCT tissue samples (25 primary tumors and 10 metastatic samples) and four (T)GCT cell lines (TCam-2, NCCIT, NTera-2, and 2102Ep, previously characterized by us, including copy number alterations [34], and cultured as described previously [30,35]) were selected for high-resolution methylation profiling with Illumina’s EPIC array (see below).

The use of patient samples was approved for research by the Medical Ethical Committee of the EMC (the Netherlands), permit no. 02.981. Samples were used according to the “Code for Proper Secondary Use of Human Tissue in The Netherlands” developed by the Dutch Federation of Medical Scientific Societies (FMWV, version, 2002; update 2011, Rotterdam, The Netherlands) and Human Research Ethics Approval 2011000572 (UQ, Australia).

### 4.2. ELISA

Human Cripto-1 DuoSet ELISA Development kit (DY145; R&D Systems Minneapolis, MN, USA) was used as per manufacturer’s instructions. Whole serum was diluted 1:2, and seminal plasma diluted 1:10 in 0.1% BSA/PBS. Standards were spiked into 0.1% BSA/PBS to generate the standard curve for interpolation. All standards and unknowns were assayed in duplicate 100 µL assays. Optical density was determined using a microplate reader (SpectraMax iD3 Multi-Mode, San Jose, CA, USA) with the 540 nm reading subtracted from the 450 nm reading. Duplicate readings for each sample were averaged, and the zero standard optical density subtracted from this value. Prism software was used to generate a four-parameter logistic curve-fit, and unknown values were interpolated to give the final CRIPTO concentration (ng/mL). THE recombinant CRIPTO standard detection range was 0 to 4000 ng/ml.

### 4.3. Methylation Profiling of the CRIPTO Promoter Using EPIC Array

After DNA extraction and bisulfite treatment of tissue samples and cell lines, they were run on the EPIC array following Illumina’s protocol (as detailed in [36]). Average methylation beta-values for the CRIPTO promoter region (hg19 coordinates chromosome 2, start: 131356624; end: 131358623) of the several samples were plotted.

### 4.4. miR-371a-3p Quantification Pipeline (Including Quality Control)

MicroRNAs were purified via magnetic bead-based isolation (TaqMan^®^ miRNA ABC Purification Bead Kit, Thermo Fisher, Carlsbad, CA, USA), following a pipeline as we previously described [31,37]. Briefly, 50 µL of serum/seminal plasma was used as starting material, and 80 µL of magnetic beads (Human Panel A Beads, Lot: 1906033/1; Ref: 4473085) was used for the isolation in an automated procedure (KingFisher Flex with 96 KF Head, Thermo Fisher Sc, cat.nr. 5400620). This was followed by cDNA synthesis using 10 µL of the purified microRNAs (TaqMan MicroRNA Reverse Transcription Kit, Thermo Fisher cat.nr. 4366597). Pre-amplification (12 cycles) was performed (2× TaqMan PreAmp Master Mix, Thermo Fisher Scientific) and quantitative reverse transcription-polymerase chain reaction (RT-qPCR) was run in QuantStudio 12K Flex Real-Time PCR System (Thermo Fisher), using the 2× TaqMan Universal Master Mix (Thermo Fisher Scientific) and the assays: ath-miR159a (assay 000338), hsa-miR-30b-5p (assay 000602), hsa-miR-20a-5p (assay 000580), and hsa-miR-371a-3p (assay 002124) (Thermo Fisher Scientific).

The non-human spike-in ath-miR-159a was included in a fixed amount (2 μL of a 1 nM stock solution) as quality control of both the miRNA isolation and cDNA synthesis. Values were normalized to the endogenous reference miR-30b-5p (for serum) and miR-20a-5p (for seminal plasma), as previously detailed [19,38], and relative levels were described and plotted as 40-ΔCt, for readability. In each plate, appropriate positive (seminoma-like cell line TCam-2 [34]) and negative (no template control) controls were included.

### 4.5. Statistical Analysis

All data were tabulated using Microsoft Excel 2016 and analyzed using GraphPad Prism 6. Patient cohorts were represented as the median and interquartile range (IQR). Percentages were calculated based on the number of cases with available data. ELISA and RT-qPCR data are presented as mean ± S.E.M within each category, and variability between categories assessed using the unpaired *t*-test. Correlations between continuous variables were assessed using Pearson’s correlation test (*r*). The Chi-square test for trend was used to compare the proportion of AFP-positive and β-HCG-positive cases according to disease stage. For reporting data on biomarker positivity (for CRIPTO and miR-371a-3p), the average concentration/relative levels in the control pool were used as the cutoff (as described in [28]). Statistical significance was set at *p* < 0.05 and is noted in each figure legend.

## 5. Conclusions

In summary, we have provided the largest sera analysis for CRIPTO (and the largest seminal plasma analysis for both CRIPTO and miR-371a-3p) to date. Our findings confirm the tractability of CRIPTO as a potential TGCT biomarker if the assay can be made more sensitive and larger cohorts can be assessed for some subgroups, particularly GCNIS-only patients. Although we postulated that semen assessment held the most promise for a sensitive diagnostic assay, limitations to our subgroup sizes greatly impeded confirmation (either positively or negatively) of its usefulness for TGCT diagnosis or prognosis. This will be an ongoing focus, along with further assessment of whether CRIPTO concentration can be a readout of fertility.

For the time being, miR-371a-3p remains the best-known biomarker for TGCT patients, as demonstrated by its sensitivity and specificity in several studies and independently validated in this work. The significant overlap in CRIPTO levels between controls and patients in the various subgroup analyses indicate that finding a relevant cohort and achieving a comparable performance will be difficult; further refinement of the assay and possibly multiplexing CRIPTO expression with other biomarkers will be instrumental in this context.

## Figures and Tables

**Table 1:** CRIPTO concentration, according to histology in the serum cohort.

All Sample Types	No. Positive Samples/Total	(CRIPTO) ng/mL		*p*-Value *
		Mean (SEM)	Range	
Control	30/48	0.79 (0.29)	0.0–10.52	-
Seminoma	15/27	1.26 (0.43)	0.0–11.27	0.3258
Non-Seminoma	134/190	1.59 (0.16)	0.0–11.55	0.0297
GCNIS	5/5	0.93 (0.38)	0.0–1.83	0.8764
Non-Seminomas Only				
Control	30/48	0.79 (0.29)	0.0–10.52	-
Mixed	104/147	1.58 (0.19)	0.0–11.27	0.0309
EC	24/32	1.76 (0.38)	0.0–8.69	0.0331
YST	2/3	1.10 (0.55)	0.0–1.77	0.7748
TE	4/8	0.27 (0.12)	0.0–0.83	0.4200
Mixed Tumors Only				
Control	30/48	0.79 (0.29)	0.0–10.52	-
Mixed, EC-positive	95/132	1.58 (0.19)	0.0–11.55	0.0315
Mixed, EC-negative	9/15	1.63 (0.73)	0.0–9.93	0.1849

* *p*-values refer to the comparison between the individual patient groups and controls. Abbreviations: EC—embryonal carcinoma; GCNIS—germ cell neoplasia in situ; SEM—standard error of mean; TE—teratoma; YST—yolk sac tumor.

**Table 2:** CRIPTO concentration according to disease stage in the serum cohort.

Patient Group	No. Positive Samples/Total	(CRIPTO) ng/mL		*p*-Value *
		Mean (SEM)	Range	
NS stage I	23/58	0.94 (0.20)	0.00–8.0	-
NS stage II	9/23	2.32 (0.58)	0.0–8.84	0.0052
NS stage III	13/13	2.43 (0.81)	0.00–9.93	0.0097

* *p*-values refer to the comparison to NS stage I group; Abbreviations: NS—non-seminoma; SEM—standard error of mean.

**Table 3:** CRIPTO Concentration in Serum and Seminal Plasma Samples of Male Controls.

Control Group	No. Positive Samples/Total	(CRIPTO) ng/mL		*p*-Value *
		Mean (SEM)	Range	
Serum controls	30/48	0.79 (0.26)	0.0–10.52	0.0001
Seminal plasma controls	5/6	4.26 (1.14)	0.0–7.37	

* *p*-values refer to the comparison between both control groups (serum vs. seminal plasma). Abbreviations: SEM—standard error of mean.

**Table 4:** CRIPTO concentration and miR-371a-3p levels in seminal plasma samples.

Patient Group	No. CRIPTO Positive Samples/Total	(CRIPTO) ng/mL		*p*-Value *	miR-371a-3p Levels		*p*-Value *
		Mean (SEM)	Range		Mean (SEM)	Range	
Seminal plasma controls (normospermia)	5/6	4.26 (1.14)	0–7.4	-	29.96	24.00–34.51	-
Non-obstructive azoospermia	62/67	6.26 (0.68)	0–22.1	0.3917	28.75 (0.30)	22.43–33.99	0.1087
Obstructive azoospermia (clinically)	10/10	7.60 (2.10)	1.3–23.6	0.2653	28.33 (1.21)	21.57–34.19	0.2765
Obstructive azoospermia (CBAVD)	0/1	0	0	N/A	25.68 (0.82)	24.86–26.49	N/A
Seminal plasma controls (normospermia)	5/6	4.26 (1.14)	0–7.4	-	29.96 (0.88)	24.00–34.51	-
Non-obstructive azoospermia, JS < 4	36/38	6.55 (0.97)	0–22.1	0.3669	29.25 (0.35)	25.47–33.99	0.3725
Non-obstructive azoospermia, JS ≥ 4	24/27	5.27 (0.86)	0–17.3	0.6016	28.14 (0.43)	23.6–31.31	0.0553
GCNIS-only	1/1	3.05 (N/A)	N/A	N/A	25.61 (N/A)	N/A	N/A
TGCT	3/3	10.24 (5.17)	4.6–20.6	0.1569	27.51 (3.27)	22.43–33.61	0.3198

* *p*-values refer to the comparison between the various groups and controls. Abbreviations: CBAVD—congenital bilateral absence of vas deferens; GCNIS—germ cell neoplasia in situ; JS—Johnsen’s score; N/A—not applicable (single sample in the group); SEM—standard error of mean; TGCT—testicular germ cell tumor.

**Table 5:** Clinicopathological features of the serum cohort.

Variables	Samples (*n* = 270)
Controls (*n*)	48
Age (years (median, interquartile range))	54 (38–73)
TGCT patients (*n*)	217
Age (years (median, interquartile range))	29 (25–39)
Histologic subtypes (*n*, %)	
Pure seminoma	27/217 (12.4)
Non-seminomas	190/217 (87.6)
Pure embryonal carcinoma	32/190 (16.8)
Pure postpubertal-type yolk sac tumor	3/190 (1.6)
Pure postpubertal-type teratoma	8/190 (4.2)
Mixed tumor	147/190 (77.4)
Stage (*n*, %)	
I	58/94 (61.7)
II	23/94 (24.5)
III	13/94 (13.8)
GCNIS patients (*n*)	5
Age (years (median, interquartile range))	30 (17–46)

Abbreviations: GCNIS—germ cell neoplasia in situ; TGCT—testicular germ cell tumor.

**Table 6:** Clinicopathological features of the seminal plasma cohort.

Variables	Samples (*n* = 94)
Normospermia (*n*)	15
Age (years (median, interquartile range))	38 (33–43)
Volume (mL (median, interquartile range))	3.7 (2–4)
Concentration (10^6^/mL (median, interquartile range))	41 (29–75)
Motility (% (median, interquartile range))	47 (38–56)
VCM (median, interquartile range)	60 (33–153)
Azoospermic patients (*n*)	79
Age (years (median, interquartile range))	35 (31–40)
Azoospermia type (*n*, %)	
Obstructive azoospermia (CBAVD)	2/79 (2.5)
Obstructive azoospermia (epididymal obstruction) *	10/79 (12.7)
Non-obstructive azoospermia #	67/79 (84.8)
TESE positive (*n*, %)	46/79 (58.2)
Johnsen’s score (*n*, %)	
2	41/79 (51.9)
3	1/79 (1.3)
4	2/79 (2.5)
5	2/79 (2.5)
6	1/79 (1.3)
7	3/79 (3.8)
8	8/79 (10.1)
9	15/79 (19.0)
10	6/79 (7.6)

* Includes one patient with a TGCT; # Includes two patients with a TGCT, one with GCNIS-only, and three with *AZF* deletion; Abbreviations: CBAVD—congenital bilateral absence of vas deferens; GCNIS—germ cell neoplasia in situ; TESE—testicular sperm extraction; TGCT—testicular germ cell tumor; VCM—volume × concentration × %motility.

## Supplementary Materials

The following are available online at https://www.mdpi.com/2072-6694/12/3/760/s1, Figure S1: Methylation levels for CRIPTO promoter derived from EPIC array analyses on cell lines (A), primary tumor tissues (B) and metastatic tumor tissues (C). Average beta values are plotted. Abbreviations: EC—embryonal carcinoma; GCNIS—germ cell neoplasia in situ; SE—seminoma; TE—teratoma; YST—yolk sac tumor; Figure S2. AFP (A and C) and β-HCG (B and D) levels according to disease stage. Levels of AFP and β-HCG according to disease stage (A and B; error bars represent mean ± S.E.M); number of positive cases according to disease stage (C and D). Abbreviations: AFP—alpha fetoprotein; β-HCG—human chorionic gonadotropin subunit beta; Figure S3. Correlation between years of sample storage and (A) CRIPTO concentration and (B) miR-371a-3p relative levels in the non-seminoma serum cohort. Relative levels of miR-371a-3p were normalized with miR-30b-5p and plotted in 40-ΔCt format; Supplementary Table S1: CRIPTO concentration analyses presented in Table 2, with outlier value in the control group removed.
