# Supplementary material for: CRIPTO and miR-371a-3p Are Serum Biomarkers of Testicular Germ Cell Tumors and Are Detected in Seminal Plasma from Azoospermic Males
Source: Cancers (Basel). 2020 Mar 23;12(3):760. doi: 10.3390/cancers12030760 (PMC7140045; doi:10.3390/cancers12030760)
Supplement: Supplementary file 1 [file cancers-12-00760-s001.pdf]

cancers

Article

CRIPTO and miR-371a-3p Are Serum Biomarkers of Testicular Germ Cell Tumors and Are Detected in Seminal Plasma from Azoospermic Males

Cassy M. Spiller, João Lobo, Willem P. A. Boellaard, Ad J. M. Gillis, Josephine Bowles, and Leendert H. J. Looijenga

Supplementary Materials

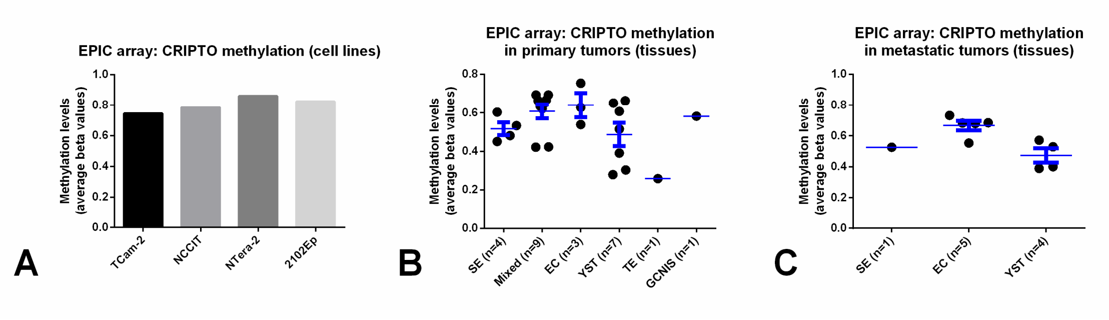

Figure S1. Methylation levels for CRIPTO promoter derived from EPIC array analyses on cell lines (A), primary tumor tissues (B) and metastatic tumor tissues (C). Average beta values are plotted. Abbreviations: EC – embryonal carcinoma; GCNIS – germ cell neoplasia in situ; SE – seminoma; TE – teratoma; YST – yolk sac tumor.

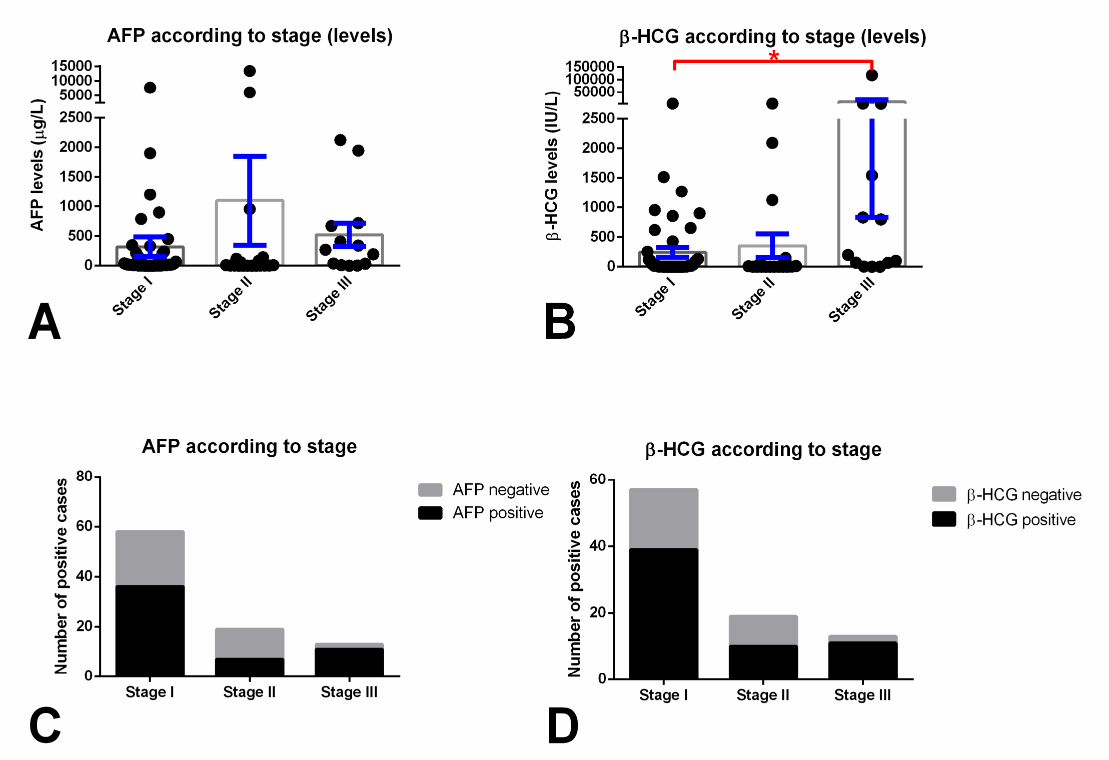

Figure 2. AFP (A and C) and β-HCG (B and D) levels according to disease stage. Levels of AFP and β-HCG according to disease stage (A and B; error bars represent ±S.E.M); number of positive cases according to disease stage (C and D). Abbreviations: AFP – alpha fetoprotein; β-HCG – human chorionic gonadotropin subunit beta.

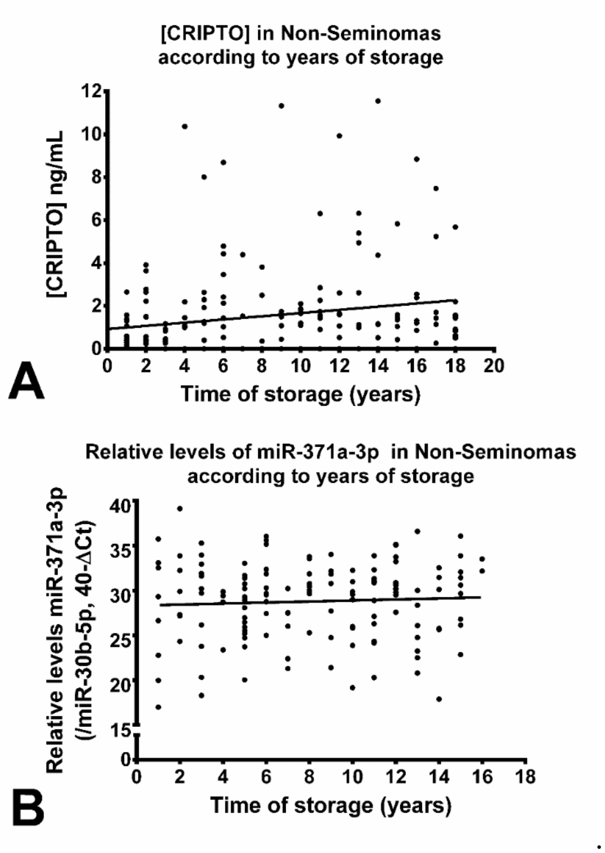

Figure 3. Correlation between years of sample storage and A) CRIPTO concentration and B) miR-371a-3p relative levels in the non-seminoma serum cohort. Relative levels of miR-371a-3p were normalized with miR-30b-5p and plotted in 40-ΔCt format.

Table S1. CRIPTO concentration analyses presented in Table 2, with outlier value in the control group removed.

**All Sample Types**	***p*-value***
Control	-
Seminoma	0.2700
Non-Seminoma	0.0042
GCNIS	0.5157
Non-Seminomas Only	*p*-value*
Control	-
Mixed	0.0045
EC	0.0022
YST	0.4397
TE	0.4288
Mixed Tumors Only	*p*-value*
Control	-
Mixed, EC-positive	0.0043
Mixed, EC-negative	0.0390

* *p*-values refer to the comparison between the individual patient groups and controls. Abbreviations: EC – embryonal carcinoma; GCNIS – germ cell neoplasia *in situ*; TE – teratoma; YST – yolk sac tumor.
